# Social disparities in body mass index (BMI) trajectories among Chinese adults in 1991–2011

**DOI:** 10.1186/s12939-017-0636-5

**Published:** 2017-08-16

**Authors:** Changchun Fang, Ying Liang

**Affiliations:** School of Social and Behavioral Sciences, Nanjing University, 163 Xianlin Avenue, Qixia District, Nanjing, 210023 Jiangsu Province People’s Republic of China

**Keywords:** Body mass index trajectories, Obesity and overweight, Chinese adults, Socio-economic status, China health and nutrition survey, Hierarchical linear model

## Abstract

**Background:**

Obesity is a serious public health problem in China. The relationship between obesity and socio-economic status (SES) is changing and affected by uncertainty, particularly, in developing countries. The sex-related differences in body mass index (BMI) trajectories are controversial and require substantial empirical data for updating and enriching.

**Methods:**

This study examined the relationship between SES and BMI in Chinese adults from a dynamic perspective using longitudinal data (1991–2011) from the China Health and Nutrition Survey (CHNS). Then, sex-related differences were determined. A hierarchical linear model was used.

**Results:**

SES positively affected the male BMI changes, with faster BMI growth rates in the high-SES males over the past 20 years. By contrast, female BMI was only affected by BMI baseline and residential area. Specifically, greater BMI baseline led to greater BMI growth rate and earlier BMI decline. In the past 20 years, the BMI growth rate has been greater in the urban females than in the rural females.

**Conclusions:**

The relationship between SES and obesity is complex in China, and a substantial sex-related difference exists. We argue that this large sex-related difference is due to the rapid economic and social changes that have affected national health and increased the gender inequality and social role restrictions in females. We provide insights for further research and policy recommendations.

## Background

Obesity is a pandemic. Given people’s diet changes and reduced physical activities, obesity is no longer a sole problem of developed countries but also of developing countries, including China [1, 2]. With its large population base and rapid growth rate, China has the world’s largest population of obese people. According to a study’s estimate, China had approximately 89.6 million obese people (43.2 million males, 46.4 million females) in 2014 [3].

The obesity and overweight epidemic is affected by both environmental and personal factors. On one hand, external socio-cultural factors, such as food supply, diet culture, and behavioral patterns, affect a person’s weight [4, 5]. Fast food diet and sedentary behavior may lead to obesity and overweight status. On the other hand, obesity and overweight are affected by many factors, including genes, individual choice [4], obesity self-awareness [6], and personal dietary preferences [7]. Various factors jointly shape a person’s way of life and hence affect the person’s weight.

Serious obesity and overweight problems negatively affect a person’s health. Overweight or obese people manifest deteriorating physical indicators and suffer from various high-risk diseases [8]. Overweight or obese people are more prone to develop prehypertension and hyperglycemia [9, 10] than the general population. Recent studies have shown that adult men with obesity and overweight problems have a lower sleep quality than those of adult men in other body mass index (BMI) groups [11]. Obesity also worsens children’s cardio-metabolic health in their adulthood [12] and increases comorbidities [13]. Obesity or overweight status may affect a person’s mental health. Researchers reviewed medical papers on obesity and mental disorders from 1966 to 2003 and found possible associations between the two [14]. An obese person tends to be stigmatized and may hence perceive a low body image, hold a low self-esteem, suffer from high stress and anxiety levels [15], and possess an increased risk of depression and other psychological problems [16]. Given the increasing problem and the serious consequences of obesity, China’s obesity problem must be further investigated.

The prevalence of obesity and overweight has resulted in social disparity because of the effects of socio-economic conditions. Socio-economic status (SES) factors, such as education, occupation, and income, most commonly lead to the disparities in obesity and overweight issues [17, 18]. However, the effects differ among countries of different developmental levels. The influence of SES on obesity and overweight is diminishing in some developed countries [19]. For example, in the United States, the influence of personal characteristics is weakening, whereas that of social-environmental factors is strengthening [20]. This correlation is becoming increasingly complex in developing countries. As a country’s economic development increases, obesity less likely exclusively affects people of high SES [21].

The impact mechanism underlying the sex-related differences in BMI changes is also complex. Chile, Brazil, Peru, and other countries that have experienced “growth miracles” similar to that of China reported an inverse relationship between BMI and SES in females. However, some East Asian countries, such as India, showed a strong positive relationship between SES and BMI [22]. By contrast, 1993 data from China revealed a heightened obesity likelihood among people of high SES [23]. In another study based on surveys in 1997, a weakening relationship between high SES and obesity in China was observed [21]. Unfortunately, these data are relatively outdated. China has achieved significant economic development in the past few decades, and its society has undergone tremendous changes. Thus, whether the relationship between SES and obesity has changed over time is worth exploring.

A dynamic perspective must be used when investigating obesity and social disparity. BMI is generally used to determine an individual’s obesity or overweight status. Longitudinal data are useful for identifying the social disparities in BMI trajectories. Analyzing these data to obtain long-term information on BMI was proven beneficial to our analysis of historical BMI changes and BMI trend prediction. Longitudinal data have been used extensively in analyzing BMI trajectories [17, 24]. Longitudinal study has been employed to explore the relationship between demographic characteristics and BMI. For example, American researchers utilized the National Health and Nutrition Examination Survey data from 1971 to 2002 to analyze the BMI of different races in the United States [25]. The relationship between SES and BMI varied among different races. However, the findings focusing on racial factors may not apply to non-immigrant countries. Chinese researchers adopted the China Health and Nutrition Survey (CHNS) from 1989 to 2006 to study the biomorphic trajectory of Chinese BMI; this study emphasized the significant disparity in females [18]. However, this research did not focus on the relationship between SES and BMI. BMI inequality is slowly being reflected by a developing social economy. This phenomenon can be observed particularly in China, which has experienced rapid economic development and significant social change. Thus, the empirical data for studying the impact factors of BMI must be updated to determine the implications of or offer advice as regards the national health problem, public health decision-making, and medical resource allocations.

Sex-related differences in the relationship between SES and obesity are substantial and must hence be given close attention [19, 26–29]. In China, findings on sex-related differences are inconsistent. A survey in 2007 showed that the prevalence of obesity was higher in males (10.6%) than in females (8.8%) [29]. By contrast, the 2014 global epidemiological data from China revealed an opposite relationship, with 43.2 million obese men and 46.4 million obese women [3]. The females’ obesity status is prominent and fuels this health inequality situation. In developing countries, such as China, Brazil, and South Africa, the sex-related differences in the relationship between SES and BMI are substantial (positive and inverse relationships in males and females, respectively) [21]. However, the data used in the above study were obtained from eight provinces in China in 1997 and are relatively obsolete. Additional epidemiological data are necessary to update the findings.

Using longitudinal data from 1991 to 2011, this study examined the effects of SES on people’s BMI growth curves in the past 20 years and compared the sex-related differences. This study not only aims to re-examine the relationship between social status and people’s obesity or overweight problems under China’s rapid social changes background, but also to update added empirical data from developing countries to the international arena. Finally, we hope that this work can draw public attention toward the health issue. The research results intend to provide useful suggestions for public health decision-making or medical resource allocation.

## Methods

### Data and measures

Data were collected from the China Health and Nutrition Survey (CHNS), an international collaborative project between the Carolina Population Center at the University of North Carolina at Chapel Hill and the National Institute for Nutrition and Health at the Chinese Center for Disease Control and Prevention. The CHNS was designed to examine how the social and economic transformation of the Chinese society has affected the health and nutritional status of its population. The project description of CHNS and the survey procedures have been described in detail in other studies [30, 31].

CHNS was conducted in 1989, 1991, 1993, 1997, 2000, 2004, 2006, 2009, and 2011. However, the 1989 survey did not use the same standardized procedures or scales of the subsequent survey years. Thus, we utilized the data from the later eight waves of observation and set the observation from 1991 as the baseline. The people aged above 45 years old in 1991 were older than 65 years old in 2011. Their indices also easily varied because of aging-related factors, such as physiological degeneration, physical activity, and basal metabolic rate, as well long-term diseases [32]. More importantly, the respondents aged over 45 years old at baseline more likely dropped out from the investigation than the younger respondents because of death and other reasons in subsequent years. The sample loss caused by death was health-related and should be avoided. To reduce the negative influence of this type of sample loss, we chose the individuals aged between 18 and 45 years old at baseline. Thus, the male age at 18–45 years old was set as the baseline for the participants (2496 participants in 1991). We obtained 2496 male samples at baseline, 1811 in 1993, 1245 in 1997, 1237 in 2000, 999 in 2004, 938 in 2006, 956 in 2009, and 906 in 2011 for a total of 10,588 observations. The number of repeat measures for participants ranged from two to eight. Female samples were also obtained from the CHNS in 1991–2011. We obtained 2494 female samples in 1991, 1893 in 1993, 1243 in 1997, 1253 in 2000, 1012 in 2004, 944 in 2006, 855 in 2009, and 836 in 2011 for a total of 10,530 samples. The data of pregnant women in their particular gestation periods were removed.

The primary dependent variable BMI was calculated as weight (kg) divided by height square (m^2^). For every individual, all BMI measurements from 1991 to 2011 were utilized to estimate the BMI trajectory in the past 20 years. Time (T) was measured by calendar year, and the value of T is equal to the year of interview minus 1991 (time point of baseline survey). Thus, the T of the baseline is “0”. Socioeconomic status (SES) was measured by education and “total net individual income” at the baseline survey.^1^ Education (the variable was named “Edu” in the models) was recoded as 1 (>9 years of formal education) or 0 (≤9 years of formal education). Income (the variable was named “income” in the models) was determined by using logarithm. Considering the difference between and rural and urban areas, the type of household register in China could indicate people’s SESs to some extent. We also used region (urban = 1, rural = 0) as an indicator. The baseline BMI (BMI_0_) was set as the control variable.

### Statistical analysis

Every individual possesses a BMI trajectory, and this study aimed to identify the effects of individual SES on the person’s BMI trajectory. The BMI trajectory is within the person, whereas the difference in SES characteristic is among persons. Accordingly, we conducted two-level hierarchical linear models (HLM) in our study [33, 34]. Each individual involves one to eight unequal observations, which are embedded in the individual. Thus, we adopted HLM.

The BMI of each individual may change over time, and the change is affected by individual characteristics. The time-varying changes in BMI caused by unobserved individual characteristics may be understood as the random variances of the individual (our model considered these changes as random effects). We also assumed that the time-varying changes in the BMIs of the individuals with similar SES characteristics are fixed (our model consider these changes as fixed effects). This study focused on whether the BMI changes of different individuals would reflect the individuals’ SES differences.

The time-related variances in individuals’ BMIs are inherent in the process of individual change, which also differs because of different individual SES characteristics. Therefore, using a two-level HLM analysis is appropriate. The equations of levels 1 and 2 are indicated below. Level 1 reflects how one’s BMI varies over time, whereas level 2 shows the difference in BMI changes between different individuals over time.

In level 1, the trajectory of weight gain not only changes but may also accelerate over time. Therefore, the trajectory model within a person is as follows:

Level 1: (within person)

1 $$ {BMI}_{jt}={\beta}_0+{\beta}_1T+{\beta}_2{T}^2+r $$

In level 2, we added personal socioeconomic characteristics to explain the coefficient variation of in the Eq. (1) of level 1. We obtained the models as follows:

Level 2: (between persons)

2 $$ {\beta}_0={\gamma}_{00}+{\gamma_{01}}^{\ast } age+{\gamma_{02}}^{\ast }{BMI}_0+{\gamma_{03}}^{\ast } Edu+{\gamma_{04}}^{\ast } income+{\gamma_{05}}^{\ast } urban+{\mu}_0 $$

3 $$ {\beta}_1={\gamma}_{10}+{\gamma_{11}}^{\ast } age+{\gamma_{12}}^{\ast }{BMI}_0+{\gamma_{13}}^{\ast } Edu+{\gamma_{14}}^{\ast } Lnincome+{\gamma_{15}}^{\ast } urban+{\mu}_1 $$

4 $$ {\beta}_2={\gamma}_{20}+{\gamma_{21}}^{\ast } age+{\gamma_{22}}^{\ast }{BMI}_0+{\gamma_{23}}^{\ast } Edu+{\gamma_{24}}^{\ast } income+{\gamma_{25}}^{\ast } urban+{\mu}_2 $$

where *β*_0_ is the average variances level in people’s BMIs over time, *β*_1_ is the effect of time on the BMI coefficient or the degree of change (rate) in people’s BMIs over time, and *β*_2_ is the influence coefficient of time square on BMI. In other words, the last variable is the change in people’s BMIs with accumulative time change.

Equations (2) and (3) take the coefficients in eq. (1) as dependent variables and personal socioeconomic charateristics as predictors to explain the BMI changes differences between individuals. Equation (3) was designed to predict the variation of the speed (*β*_1_ in Eq. 1) of BMI changes with time, and Eq. (4) was designed to predict the cumulation (*β*_2_ in Eq. 1) of BMI changes with time.

## Results

### Sample characteristics

The descriptive statistics for variables at levels 1 and 2 are shown in Table 1. Table 1 shows that 2496 men aged 18–45 years old were included in the study since 1991, and the total number of years yielded 10,588 observations. The mean BMI was 22.34, and 22% of the population attained >9 years of formal education. The mean income (logarithmic) was 8.04, and 29% lived in urban areas. The average age of the sample was 31.68 years old in the base period (1991). By contrast, 2494 women aged 18–45 years old were included, and the cumulative number of years yielded 10,530 observations. The mean BMI was 22.82, and 17% of the population attained >9 years of formal education. The mean income (logarithmic) was 7.91, and 28% lived in urban areas. The average age of the sample was 31.82 years old in the base period (1991).

**Table 1:** descriptive statistics for variables at level 1 and level 2

	Variable	*N*	Mean	SD	Minimum	Maximum
	Level-1 variables					
Male	BMI	10,588	22.34	2.91	12.68	57.01
	T(time)	10,588	7.99	7.06	0.00	20.00
	T^2^	10,588	113.74	135.41	0.00	400.00
Female	BMI	10,530	22.82	3.18	9.03	51.93
	T(time)	10,530	7.78	6.97	0.00	20.00
	T^2^	10,530	109.08	132.47	0.00	400.00
	Level-2 variables					
Male	age	2496	31.68	7.80	18.00	45.00
	BMI_0_(baseline BMI)	2496	21.35	2.37	14.74	32.39
	Edu	2496	0.22	0.42	0.00	1.00
	Income	2496	8.04	0.93	0.99	11.10
	urban/rural	2496	0.29	0.46	0.00	1.00
Female	age	2494	31.82	7.69	18.00	45.00
	BMI0(baseline BMI)	2494	21.69	2.66	13.95	40.34
	Edu	2494	0.17	0.37	0.00	1.00
	Income	2494	7.91	0.89	2.15	10.58
	urban/rural	2494	0.28	0.45	0.00	1.00

Before conducting multivariate analysis, we used descriptive statistics to show how the BMI varied over time in relation to socioeconomic characteristics in selected age groups. The results are shown in Tables 2 and 3. Among the male age groups born in 1946–1960, those with lower educational levels (Edu = 0) achieved higher rates of BMI growth in the last 20 years than those of the control of other factors under the same age groups (Table 2). On the contrary, the age groups born after 1961 with higher educational levels reported higher BMI growth rates. For the women, those with lower educational levels displayed faster BMI growth rates in all the age groups.

**Table 2:** The changes of BMI over time vary with education for selected age groups

Male	Edu**	1991	1993	1997	2000	2004	2006	2009	2011	Δ(%)*
1946–1950	1	22.64	22.52	23.39	23.65	23.43	23.52	24.78	23.98	5.92
	0	21.68	21.91	22.17	22.59	22.64	22.57	22.88	23.09	6.51
1951–1955	1	22.34	22.66	23.73	24.25	24.01	23.99	24.05	23.68	6.04
	0	21.64	21.89	22.19	22.31	22.61	22.63	23.06	23.38	8.01
1956–1960	1	21.92	22.18	22.66	23.22	23.75	24.14	23.74	23.61	7.73
	0	21.48	21.61	22.06	22.7	22.92	23	23.64	23.91	11.28
1961–1965	1	21.36	21.66	22.72	23.5	23.25	23.35	24.54	24.52	14.82
	0	21.27	21.54	21.81	22.48	23	23.23	23.67	24.04	13.03
1966–1973	1	21.05	21.86	22.27	23.09	23.19	23.24	24.01	24.80	17.82
	0	20.48	20.82	21.32	21.79	22.38	22.48	22.98	23.19	13.22
Female	Edu	1991	1993	1997	2000	2004	2006	2009	2011	Δ(%)
1946–1950	1	24.05	24.73	24.39	23.79	24.81	24.94	25.13	24.52	1.95
	0	22.59	22.84	23.05	23.38	23.59	23.46	23.52	23.60	4.47
1951–1955	1	21.57	21.60	22.31	22.82	22.72	22.85	22.24	22.08	2.36
	0	22.11	22.30	22.70	23.20	23.44	23.65	23.82	23.88	8.01
1956–1960	1	21.43	21.59	22.45	22.80	22.50	23.00	23.34	23.71	10.64
	0	22.11	22.37	22.92	23.70	23.90	24.30	24.56	24.66	11.53
1961–1965	1	20.90	21.22	21.90	22.87	22.13	22.15	22.72	24.10	15.31
	0	21.37	21.74	22.29	22.97	23.44	23.58	24.22	24.75	15.82
1966–1973	1	20.21	20.19	21.74	21.93	23.29	23.18	22.08	22.13	9.50
	0	21.08	21.16	21.79	22.24	22.95	22.92	23.02	23.61	12.00

Note:

$$ \Delta ={\frac{BMI_{2011}-{BMI}_{1991}}{BMI_{1991}}}^{\ast }100\% $$.

* 1 to 2011 BMI growth rate

** Edu = 1 means more than 9 years of formal education, and Edu = 0 means 9 or less than 9 years of formal education

**Table 3:** The changes of BMI over time vary with urban/rural for selected age groups

Male		1991	1993	1997	2000	2004	2006	2009	2011	∆(%)
1946–1950	rural	21.51	21.76	22.09	22.59	22.62	22.50	22.98	23.11	7.44
	urban	22.65	22.69	23.13	23.54	23.53	23.89	24.07	23.84	5.25
1951–1955	rural	21.46	21.72	22.09	22.39	22.50	22.64	22.95	23.14	7.82
	urban	22.49	22.82	23.89	24.26	25.32	24.46	25.07	24.91	10.77
1956–1960	rural	21.67	21.82	22.13	22.71	23.03	23.21	23.53	23.65	9.11
	urban	21.51	21.76	22.64	23.42	24.20	24.48	24.31	24.58	14.25
1961–1965	rural	21.06	21.37	21.77	22.52	22.91	23.10	23.75	23.98	13.86
	urban	21.78	22.11	23.08	23.89	23.80	24.19	24.85	25.01	14.83
1966–1973	rural	20.43	20.76	21.13	21.61	22.29	22.39	22.80	23.15	13.34
	urban	21.01	21.74	23.00	24.28	24.28	24.08	25.08	25.86	23.07
Female		1991	1993	1997	2000	2004	2006	2009	2011	∆(%)
1946–1950	rural	22.36	22.68	22.79	23.27	23.50	23.36	23.60	23.39	4.60
	urban	23.60	23.84	24.49	24.28	24.76	24.81	23.39	25.59	8.41
1951–1955	rural	21.91	22.08	22.51	22.99	23.20	23.37	23.62	23.79	8.59
	urban	22.38	22.59	23.23	23.93	24.34	24.59	24.07	23.11	3.24
1956–1960	rural	21.80	22.05	22.58	23.33	23.71	23.96	24.32	24.48	12.34
	urban	22.29	22.58	23.63	24.45	23.38	24.56	24.41	24.48	9.86
1961–1965	rural	21.41	21.75	22.10	22.96	23.42	23.51	24.04	24.77	15.70
	urban	20.92	21.25	22.50	22.90	21.77	22.17	23.84	23.81	13.83
1966–1973	rural	21.09	21.19	21.82	22.19	22.97	22.93	23.00	23.70	12.39
	urban	20.57	20.42	21.64	22.38	23.03	23.02	22.44	22.41	8.99

In all the age groups apart from those born in 1946–1950, BMI growth was faster in the urban men than in the rural men (Table 3). A similar pattern was observed in the female respondents.

The BMI of the middle-income earners was lower than those of high-income and low-income earners, and the low-income earners showed the highest BMI (Table 4). The BMI growth rate did not considerably differ between the low- and high-income males in the 1946–1961 age group. By contrast, a substantial difference in BMI growth rate was noted between the middle- and high-income males born after 1961. For the women born in 1946–1950 and 1961–1965, the middle-income earners achieved the greatest BMI increases. For the women born in 1951–1955, the middle-income earners showed the least BMI increases. For the women born in 1956–1960, the high-income earners achieved the smallest BMI increase. For the women born in 1966–1973, high-income earners reported the largest BMI increases.

**Table 4:** The changes of BMI over time vary with income for selected age groups

Male	Income	1991	1993	1997	2000	2004	2006	2009	2011	∆(%)
1946–1950	low	21.28	21.64	21.68	22.55	22.25	22.84	23.63	24.25	13.95
	middle	22.25	22.26	22.53	22.50	22.57	22.49	22.44	23.37	5.03
	high	22.10	22.77	22.52	23.26	23.47	22.90	23.40	23.01	4.13
1951–1955	low	21.42	21.89	22.17	22.22	22.38	22.03	23.38	23.11	7.87
	middle	22.02	22.04	22.39	22.78	22.51	22.31	22.63	22.49	2.12
	high	22.21	22.60	23.26	23.21	23.88	23.72	23.57	23.72	6.8
1956–1960	low	21.25	21.60	21.72	21.75	22.26	22.14	23.43	23.80	12
	middle	21.80	21.95	22.47	22.69	22.39	23.31	22.73	23.05	5.73
	high	22.61	22.00	22.47	23.47	24.42	23.88	23.92	23.95	5.89
1961–1965	low	21.19	21.64	21.58	21.42	22.47	22.68	23.88	24.87	17.38
	middle	21.38	21.48	22.21	22.79	22.68	23.55	23.30	22.69	6.1
	high	21.56	21.61	22.61	23.61	23.58	23.26	24.14	24.29	12.67
1966–1973	low	20.49	21.04	21.07	21.77	21.62	21.26	22.78	24.55	19.82
	middle	20.90	20.83	21.61	21.81	22.73	22.14	22.58	22.94	9.79
	high	20.42	21.25	22.31	22.64	23.11	23.13	23.31	23.46	14.87
Female	income	1991	1993	1997	2000	2004	2006	2009	2011	∆(%)
1946–1950	low	22.33	22.32	22.08	22.61	22.98	22.55	23.02	23.26	4.17
	middle	22.70	23.18	23.00	23.51	23.12	23.62	23.90	24.39	7.47
	high	22.82	23.11	23.66	23.72	24.24	24.07	23.72	23.51	3.03
1951–1955	low	21.78	21.83	22.23	22.86	23.26	23.46	23.40	23.82	9.35
	middle	21.96	22.05	22.68	23.07	23.08	23.44	23.53	23.30	6.14
	high	22.26	22.58	22.95	23.41	23.65	23.72	24.02	23.89	7.33
1956–1960	low	21.77	21.97	22.52	23.08	23.50	24.06	24.07	24.56	12.82
	middle	22.00	22.31	22.88	23.61	23.39	24.08	24.68	24.48	11.28
	high	22.01	22.24	22.94	23.84	24.17	24.01	24.24	24.41	10.88
1961–1965	low	21.02	21.25	22.20	22.78	22.93	23.56	23.71	24.47	16.37
	middle	21.51	21.89	22.50	23.08	23.24	23.14	24.49	25.11	16.76
	high	21.19	21.63	21.92	22.99	23.62	23.22	24.02	24.44	15.33
1966–1973	low	21.09	21.11	21.70	22.33	22.83	23.37	23.19	23.70	12.34
	middle	20.95	21.00	21.94	22.21	23.11	22.75	23.09	23.51	12.20
	high	20.52	20.77	21.79	21.97	23.18	22.21	22.28	23.22	13.16

Notably, the above-mentioned description did not account for the growth process of individual BMI curves but only described the BMI changes over 20 years (1991–2011), particularly, the difference between the two time periods (2011 and 1991). This approach cannot accurately describe the actual BMI trajectory. In fact, the BMI trajectory is a quadratic function of time (Eq. (1)); hence, the time-related changes is an open downward curve. People’s BMIs usually grow over time, but the rise gradually slows down eventually [34]. Therefore, considering only the changes in two separate years may inaccurately estimate people’s BMI changes over time. For example, the BMI of the elderly may have experienced rapid decline due to health degradation. Therefore, we used the HLM method to examine individual BMI trajectory changes over time.

### HLM results

The study results described above are based on the analysis of differences and changes in people’s BMI with specific SES characteristics. In the following analysis, we used the HLM model to analyze how demographic characteristics affect people’s BMI changes. The results are shown in Table 5.

**Table 5:** Results of Hierarchical linear model

	Model 1 (Male)	Model 2 (Male)	Model 3 (Male)	Model 4 (Female)	Model 5 (Female)	Model 6 (Female)
Fixed effects	Estimated coefficient (Standard Error)					
β_0_						
γ_00_:intercept	21.366635 (0.046718)***	19.439828 (0.179802)***	0.906344 (0.127572)***	21.679477 (0.052926)***	19.280736 (0.207830)***	0.392720 (0.122053)***
γ_01_ : age		0.060650 (0.005794)***	0.001941 (0.001342)		0.075230 (0.006734)***	0.001771 (0.001444)
γ_02_ : BMI_0_			0.964868 (0.005054)***			0.971308 (0.004104)***
γ_03_ : Edu			−0.006151 (0.026697)			0.010334 (0.030745)
γ_04_ : income			−0.025078 (0.010421)*			0.019315 (0.012029)
γ_05_ : urban			−0.028072 (0.023644)			−0.005214 (0.025541)
β_1_						
γ_10_:intercept	0.160326 (0.008958)***	0.287446 (0.039044)***	0.596288 (0.107234)***	0.174801 (0.008880)***	0.133414 (0.047592)***	0.349757 (0.106408)
γ_11_ : age		−0.003930 (0.001163)***	−0.003072 (0.001212)*		0.001158 (0.001372)	0.002269 (0.001432)
γ_12_ : BMI_0_			−0.029210 (0.004580)***			−0.014896 (0.003761)***
γ_13_ : Edu			0.084286 (0.022512)***			0.006427 (0.025568)
γ_14_ : income			0.030222 (0.010457)***			0.007475 (0.010010)
γ_15_ : urban			0.125064 (0.023339)***			0.062926 (0.025742)*
β_2_						
γ_20_:intercept	−0.002407 (0.000487)***	−0.003085 (0.002109)	−0.014883 (0.005926)*	−0.002949 (0.000491)***	0.006691 (0.002595)**	0.001594 (0.006872)
γ_21_ : age		0.000024 (0.000064)	−0.000003 (0.000067)		−0.000281 (0.000075)***	−0.000316 (0.000078)***
γ_22_ : BMI_0_			0.001267 (0.000256)***			0.000418 (0.000260)
γ_23_ : Edu			−0.003866 (0.001172)***			−0.001397 (0.001567)
γ_24_ : income			−0.001550 (0.000578)***			−0.000242 (0.000531)
γ_25_ (urban)			−0.005359 (0.001335)***			−0.004744 (0.001625)***
Random effects	Variance component					
${\upsigma}_{\upmu_0}^2$:intercept	4.43618***	4.21466***	0.00321	5.91456***	5.58332***	0.00414
${\upsigma}_{\upmu_1}^2$:T	0.06645***	0.06571***	0.10160***	0.05323***	0.05324***	0.09682***
${\upsigma}_{\upmu_2}^2$: T^2^	0.00018***	0.00018***	0.00026***	0.00016***	0.00015***	0.00025***
${\upsigma}_{\upepsilon}^2$:Error variance	1.31591	1.31659	0.97326	1.41250	1.41327	1.06231
Number of observations	10,588			10,530		
Number of individuals	2496			2494		

Note:

Since the coefficients in the table are relatively small, this table retains multiple decimal places;

*** *P* < =0.001, **p < =0.01, *p < =0.05

**Fig. 1:**
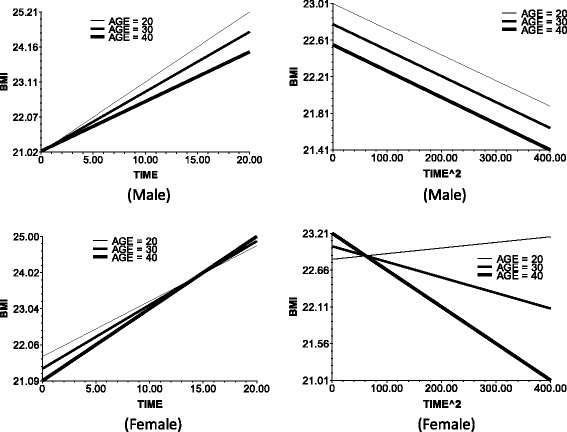
The impact of age on the slop of TIME (*left*) and TIME^2 (*right*)

**Fig. 2:**
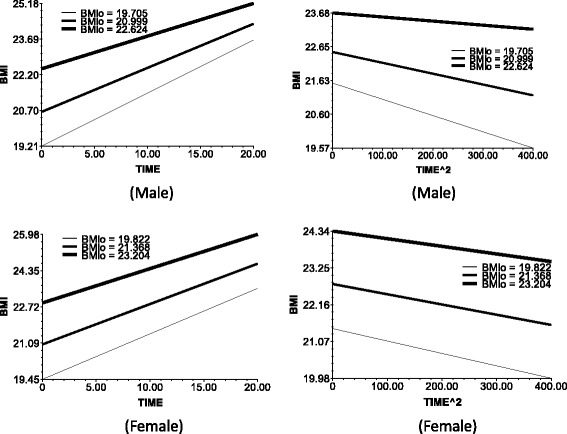
The impact of BMI_0_ (BMI at baseline) on the slop of TIME (*left*) and TIME^2 (*right*)

**Fig. 3:**
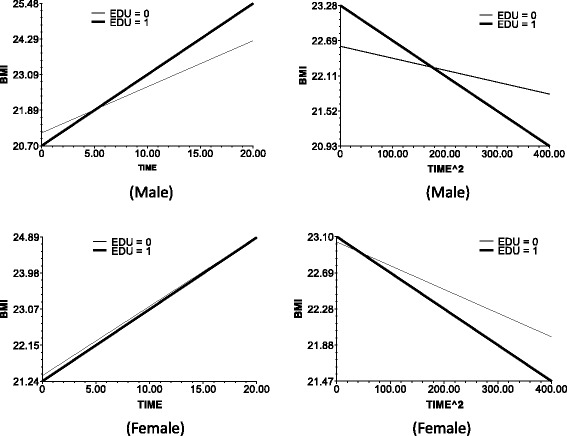
The impact of education on the slop of TIME (*left*) and TIME^2 (*right*)

**Fig. 4:**
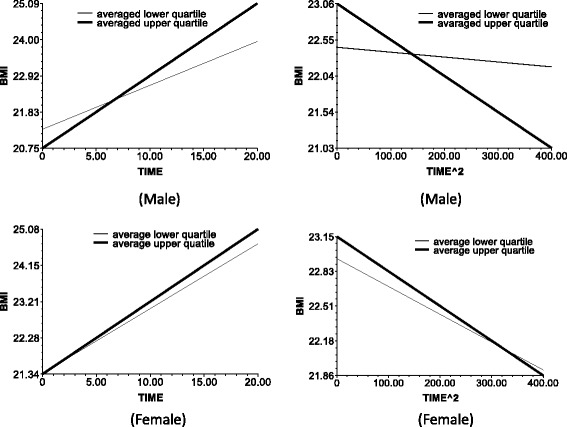
The impact of income on the slop of TIME (*left*) and TIME^2 (*right*)

**Fig. 5:**
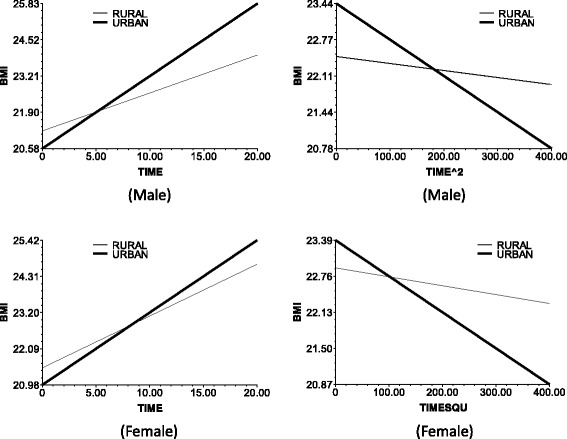
The impact of urban/rural on the slop of TIME (*left*) and TIME^2 (*right*)

Table 5 shows HLM results. Figures 1, 2, 3, 4 and 5 illustrates the effects of age, BMI_0_, education, and income on the slope of time and time square in the Level 1 model. Models 1 to 3 are for the men, and models 4 to 6 are for the women. Models 1 and 4 in Table 5 are mixed-effects models without predictors on level 2. The fixed effects of these models indicate the average changes in male/female BMI with time, and the random effects of these models show that the BMI changes with time varied between persons. We explain the variances between persons as follows.

In models 2 and 4, age was taken as predictor, and models 3 and 6 include all predictors on level 2.

1. The following data are the results in the men. Model 2 and 3 results indicate the baseline age (baseline mean age corresponds to the respondents’ birth cohort). The base period age reaction, which is the test birth cohort, or intergenerational differences negatively affect the time slope (bate 1 in Eq. 1). This observation means that the BMI growth speed was faster in the younger group than in the older in the recent 20 years. Furthermore, age did not significantly affect the BMI growth acceleration (Fig. 1). The baseline age did not significantly influence the slope of time ^ 2.
2. The BMI growth speed and growth deceleration of the individuals with higher baseline BMI scores were slower than those of the individuals with lower baseline BMI scores. The plot at the upper left corner of Fig. 2 illustrates the direct relationship of the baseline BMI with the smoothness of the time slope and the slope decline rate of time square.
3. Education positively affected the BMI growth speed. That is, the speed for the people with >9 years of school education was faster than that of the people with ≤9 years of school education. At edu = 1, the time slope is more steep (Fig. 3, upper left corner). However, the BMI growth deceleration is slower for those with low educational attainment. At edu = 0, the slope of time ^ 2 is more balanced, whereas at edu = 1, the downward slope of time ^ 2 is more steep (Fig. 3, upper right corner).
4. Income positively affected the BMI growth speed and negatively affected the BMI growth acceleration. This finding suggests that higher incomes lead to faster BMI growths and BMI growth decelerations.
5. The BMI growth speed was higher and the BMI growth deceleration was faster in the urban areas than in the rural areas. Furthermore, the time slope was steeper for the respondents of urban areas than those of rural areas (Fig. 5, upper left corner).
6. For the women, the time-varying effects differed from those of the men.
  Age significantly influenced the intercept of the female BMI growth curve but not the BMI change speed with time. Several lines almost overlap at the bottom left corner of Fig. 1. However, unlike in men, age significantly affected the acceleration of female BMI changes.
  Model 6 shows that education and income did not significantly influence the speed and acceleration of female BMI changes. For the women, the BMI change speed was mainly affected by the baseline BMI and regional residence, whereas the BMI change acceleration was affected by the age and regional residence. The male BMI was more likely affected by SES than the female BMI.

## Discussion

Previous studies reported that people with different demographic characteristics reflect different BMI patterns [17, 18, 20]. BMI changes over time is also affected by SES, and this relation is the focus of this study. This paper sampled Chinese adults by using HLM to analyze the corresponding BMI changes over the past 20 years. This study also analyzed how BMI trajectory is affected by individual demographic characteristics and compared between the impact mechanisms underlying sex-related differences in BMI trajectories.

The Chinese men with greater ages, higher education, and rural residences recorded slower BMI growths in the past 20 years. HLM results also indicated the positive effects of low educational level, high income, and urban residence on BMI trajectory. People with low baseline BMIs showed significantly higher BMI growth rates than those with higher baseline BMIs. In terms of socioeconomic variables, the Chinese men with higher educational levels were found with slower BMI growths. Traditionally, high SES is considered to correspond to a high obesity likelihood because of ready access to adequate food and the cultural preference for a fat body shape [21]. A recent study indicated that obesity is no longer considered as a disease associated solely with high-SES populations [21]. Our study showed that different SES indicators exert different effects on BMI growth, although the prevalence of obesity and overweight status decreases as educational level increases. Nevertheless, the prevalence remains relatively high among the rich people. With improved medical knowledge among the public, obesity dangers are slowly being acknowledged. Thus, men with high education pay additional attention to their diet, engage in more physicalexercises, or prevent obesity through medical service. With rapid economic development, access to adequate food is easy for the newly rich, who could then readily develop overweight or obesity problems if they lack knowledge and good health habits.

Male BMI increased more slowly in the rural residences than in the urban residences. This result may be explained by the higher physical labor working in rural males than in most urban males. Another reason may be related to the different social structures in China’s urban and rural areas. With decades of rapid urbanization in China, the economy has rapidly developed while generating some changes in people’s lifestyles, including physical activity and diet pattern [35, 36]. This phenomenon is particularly apparent in urban areas. Therefore, males become prone to increased obesity risk when they reside in urban areas and lead an unhealthy lifestyle.

The relationship between the SES and BMI in the women was complicated. This finding is inconsistent with those of some developed countries, such as England and Sweden, where SES is a social determinant of obesity severity [37, 38]. First, education and income did not significantly affect the female BMI changes. In developed countries, low educational level and occupational status are obesity risk factors in women [38]. Economic deprivation may lead to high obesity incidence among low-SES women because of difficulty in acquiring overweight control practices and healthy diet advices [39]. However, the present study, conducted in China, does not support the above-mentioned relationship. This result indicates that such characteristics do not apply to developing countries. Second, the age and place of residence significantly affected the female BMI change rate. This observation may be related to the role restriction of women in the Chinese family or society. Family status is an important influence because married women and mothers are more preoccupied than men or fathers because of housework and maintaining family relationships [40]. This social norm for women is relatively prevalent in undeveloped rural areas [41]. External environmental factors, such as long-term exposure to social media (media images depicting a thin body as ideal), could also shape a female’s perspective and control of her body image [42]. SES affects women’s BMIs by influencing their reproductive histories, unhealthy dietary habits, and psychosocial stress levels (53% of the variances) [37]. These sex-related inequality and social roles contribute to the differences in BMI changes between women and men.

Overall, this study identified the social disparities in BMI trajectories among Chinese men by using 20 years of longitudinal data. Different SES indicators revealed different relationships with BMI growth in China. Globally, people with higher SESs and living in developed countries are generally considered to possess better access to social or medical resources, as well as greater abilities to maintain a healthy diet or lifestyle, than those with lower SESs and living in developing countries. However, this pattern does not apply to China. The Chinese males with low SESs in this study experienced rapid BMI changes because of high work pressure, financial hardship, and less resources to cope with their livelihood. Women’s health was also limited by gender inequality and traditional social roles. The present findings reveal that the relationship between BMI and SES is not a simple positive or negative correlation.

Given these findings, this study suggests that public health decision-making for alleviating obesity or overweight issues should include promoting knowledge on obesity-related diseases in both high- and low-SES populations. Particularly, such educational campaign should extend to the newly rich, who will then be assisted in understanding the dangers of poor health behavior. The women, who are vulnerable to the negative impact of social inequality, deserve substantial attention and support [43, 44]. The special assistance policy provided by the government or social organizations would be helpful to ease their health issues hence improve their life quality and well-being [45, 46].

This study involved some limitations. The effects of the baseline on BMI growth were considered, but the respondents were not grouped by different overweight level. The SES measures were also diverse. Additional indicators, such as occupation and household assets, merit further attention. China must also address obesity and overweight problems in the long term. Studies on health burden and dynamic changes should be continued. Finally, other biological, social, and psychological factors, such as discrimination, self-esteem, functional limitation, and dietary habits, must not be ignored when considering the impact factors for individual BMIs. These factors may be due to obesity and overweight status, and unjust treatment may further weaken the affected individuals’ intention to control weight and cause them to surrender immediately. Future researchers can explore this topic further.

## Conclusion

Obesity in China is an urgent public health problem. With the country’s rapid economic development and social change, the BMI of the Chinese people has been affected by social disparity. The relationship between obesity and SES is also changing, and this relationship is becoming increasingly complex in developing countries. Sex-related differences are also controversial. In this study, we employed the HLM to examine the relationship between SES and BMI trajectories in a Chinese population under a dynamic perspective by using longitudinal data (1991–2011) from CHNS.

The men with better economic situations revealed faster BMI growths. This finding was likely due to the more access to adequate food of the newly rich, who could readily become overweight or obese if they lack knowledge and good health habits. The urban residents also showed greater BMI changes than those of the rural residents. It might be related to the unhealthy dietary style in urban areas. Age and place of residence significantly affected the female BMI change rate. We speculate that this influence may be related to the role of women in the Chinese family or society. Therefore, this study emphasizes that public health policy should focus not only on disadvantaged groups with low SES but also on the newly rich population. Further research attention and policy preference should be given to the female population to reduce the negative impact of social inequality on their health. Meanwhile, the BMI growth of the younger population (especially men) in the last 20 years was faster than that of the older group. This finding may be related to China’s economic development and shift from a low to a middle/high gross domestic product, which have influenced the general average lifestyle of the population.

This study provides up-to-date data on the relationship between SES and BMI in developing countries. By using longitudinal data, the study showed the dynamics of BMI trajectories in China over the past 20 years and presented details on the social disparities. China is the largest developing country with the greatest number of obese people. Hence, the obesity problem among Chinese adults and the historical changes can provide other countries with useful insights into understanding and coping with global public health problems.

## Footnotes

1 The data used were collected from individuals at multiple time points. The SES, especially the occupational status, of each individual changes over time. This feature is notable among young people. Under data pre-inspection and processing, the data will be missed if the occupation variable would be included. Thus, this study excluded the occupation variable.
